# Impact of a personalised care plan for the elderly calling emergency medical services after a fall at home: The RISING-DOM multi-centre randomised controlled trial protocol

**DOI:** 10.1186/s12877-022-02850-w

**Published:** 2022-03-04

**Authors:** Wafa Bouzid, Neda Tavassoli, Caroline Berbon, Soraya Qassemi, Vincent Bounes, Olivier Azema, Jason Shourick, Fati Nourhashémi

**Affiliations:** 1 Gérontopôle, Centre Hospitalier Universitaire de Toulouse, Toulouse, France; 2 Pôle Médecine d’Urgence, Centre Hospitalier Universitaire de Toulouse, Toulouse, France; 3 Observatoire Régional Des Urgences d’Occitanie (ORU Occitanie), Hôpital La Grave, Place Lange, 31300 Toulouse, France; 4 Unité de Soutien Méthodologique À La Recherche (USMR), Service d’Epidémiologie Clinique Et de Santé Publique, CHU de Toulouse, Toulouse, France; 5 CERPOP, UMR 1295, INSERM - Université de Toulouse III, Toulouse, France

**Keywords:** Fall, Nursing home, Elderly, Comprehensive geriatric assessment, Emergency medical service, Randomised controlled trial

## Abstract

**Background:**

A growing number of emergency calls are made each year for elderly people who fall. Many of them are not taken to hospital or are rapidly discharged from the Emergency Department (ED). Evidence shows that, with no further support, this vulnerable population is particularly at risk of injuries, dependency and death. This study aims to determine the effectiveness of a comprehensive geriatric assessment and a tailored intervention in the elderly calling on an Emergency Medical Service (EMS) for a fall at home, but not conveyed to the ED or rapidly discharged from it (less than 24 h from hospitalisation), to the time to institutionalisation or death.

**Methods:**

Rising-Dom is a two-arm randomised (ratio 1:1), interventional, multi-centre and open study. Community-dwelling elderly people (≥ 70 years) who call an EMS for a fall at home are recruited. The intervention group receives home visits by a nurse with a comprehensive fall risk assessment and a personalised intervention care plan with a planned follow-up (six nurse home visits and five nurse phone calls). Subjects enrolled in the usual care-control group continue to receive their routine care for the prevention or treatment of diseases. Primary (time to institutionalisation or death) and secondary (unscheduled hospitalisations, additional EMS calls relating to falls, functional decline and quality of life) outcome data will be collected for both groups through five phone calls made by Clinical Research Associates (CRA) blind to the participants’ group during the follow-up period (24-months). Twelve hospital centres in the South-West of France are participating in the study as study sites. The inclusion period started in October 2019 and will end in March 2022. By the end of this period, 1,190 subjects are expected to be enrolled.

**Discussion:**

Studies on elderly home falls have rarely concerned people who were not taken to hospital. The Rising-Dom intervention scheme should enhance understanding of features related to this vulnerable population and investigate the impact of a nurse care at home on delaying death and institutionalisation.

**Trial registration:**

Clinicaltrials.gov identifier: NCT04132544. Registration date: 18/10/2019. Sponsor: University Hospital, Toulouse. https://www.clinicaltrials.gov/ct2/show/NCT04132544?term=rising-dom&draw=2&rank=1

## Background

Falls are the first cause of accidental death in people over 65 and have negative physical, psychological and quality of life consequences [1]. Fall-induced deaths of the elderly are in constant increase, especially in modern societies with aging populations [2–4]. In 2016, approximately 30,000 older adults died because of a fall in the USA [5] and the overall medical spending totalled $50 billion in 2015, making falls one of the most costly health conditions among people 65 and older [6]. In addition, falls increase the risk of institutionalisation and are considered as a strong predictor of nursing home placement [7].

Home falls among elderly living in the community may require Emergency Medical Services (EMS). Studies from different countries have shown that calls for falls count for 8% to 17% of all EMS calls [8–10] with a commonly repeated use of EMS for falls [9, 11].

Although many of these subjects require transport to hospitals for examination and care, EMS data from various countries highlight that 11 to 56% of the elderly who received emergency intervention for a fall were not transported to a medical facility, often because they were not injured or refused transport [12–14]. This population is, however, particularly vulnerable and, according to some studies, half of the elderly who have fallen and called EMS had needed "unscheduled care" the month preceding the call [14, 15].

Falls are usually multifactorial and may be associated with age, impaired mobility, sensory deficits, chronic conditions, medication as well as environmental hazards [16, 17]. Numerous studies have evaluated the association between falls and intrinsic factors (for instance frailty [18], functional status [19], vision [20, 21], nutritional status [22–24], medication [25]) and extrinsic events linked to home environment risk factors (slippery floors, inadequate lighting, unstable furniture, loose rugs and carpets, etc.) [26, 27]. Considering the patient in his/her whole environment is hence a relevant issue in the identification of fall risks and the planning of efficient preventive measures [28, 29].

Few studies have focused on older people who call an EMS for a fall but are not conveyed or are rapidly discharged from ED after transportation [14]. They show that no intervention or specific care is scheduled after EMS intervention and the elderly who fall are often not treated in primary care practice [29, 30]. This population seems, however, to be particularly vulnerable with a high risk of mortality and institutionalisation. Therefore, they represent an interesting target for appropriate interventions [31, 32]. The RISING-DOM project has been designed as an answer to a critical request from our ED department to reduce resource utilisation for fall-related calls in the elderly and implementation of a fall-prevention strategy to decrease fall rates and related injuries.

In the RISING-DOM study, we intend to evaluate the subjects (70 years old and over) in their own environment by an experienced geriatric nurse whenever a fall triggers a call to the EMS (without hospitalisation or with an ED admission less than 24 h). A Personalised Intervention Care Plan (PIP) based on an initial comprehensive geriatric assessment is proposed, with targeted and prioritised actions in collaboration with the participant’s general practitioner (GP), hospital geriatrician and other health professionals if necessary. The PIP is based on the patient's desires and concerns and a maximum of three goals are established in order to increase participant's adherence.

The main purpose of the study is to assess the effect of this intervention on time to institutionalisation or death. Secondly, the impact of the PIP will be assessed regarding the number of institutionalisations or deaths, the number and time to first additional EMS call relating to a fall, the number and time to first unscheduled hospitalisation, the functional dependency level and the quality of life during follow up.

## Methods/Design

### Study design

RISING-DOM is a randomised, multi-centre, open, interventional study comparing two groups (ratio 1:1): an intervention group consisting of subjects for whom a comprehensive geriatric assessment (CGA) with a PIP and a planned follow-up are carried out at home by an experienced geriatric nurse versus a usual care control group of subjects that continue to receive their routine care for the prevention or treatment of diseases.

**Fig. 1:**
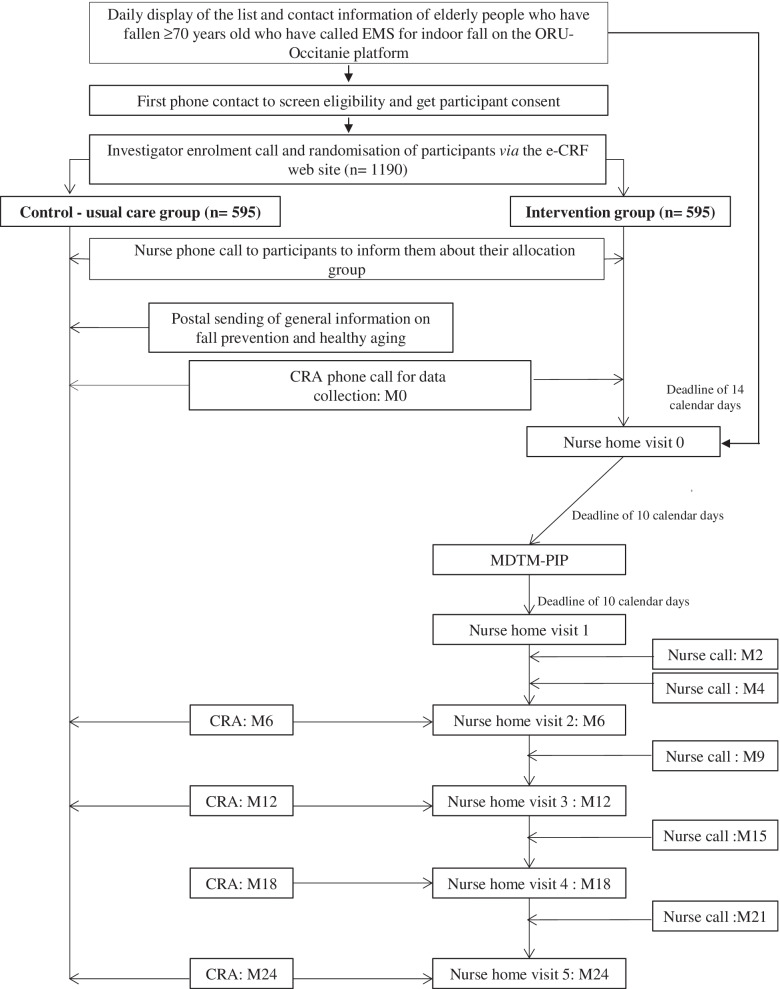
RISING-DOM study design. EMS Emergency medical service, ORU-Occitanie Observatory of Occitanie region, e-CRF electronic Case Report Form, MDTM Multidisciplinary Team Meeting, PIP Personalised Intervention care Plan, CRA Clinical research associate, M0, M2, M4, M6, M9, M12, M15, M18, M21 and M24 match months 0,2, 4, 6, 9, 12, 15, 18 and 24 respectively from the enrolment (t0) until the end of the follow-up period

RISING-DOM was designed (Fig. 1) according to a previous pilot study in which the feasibility regarding recruitment, home visits and adherence to the intervention program were tested (unpublished data). In this pilot study, carried out at our institution in 2015 in Toulouse, France (480,000 inhabitants), about 2,797 calls to an EMS for a fall were registered, among which 87% came from an elderly person aged over 70 years old. About 32% of this population called the EMS again regarding another fall in the next 6 months. Ninety-one elderly people who had fallen (mean age 83.6 years old) have been assessed; among them 43% were frail, 14% pre-frail according to Fried criteria [33] and 43% were dependent. About 58% had cognitive disorders (MMSE ≤ 24) and 49% were at risk of undernutrition (unpublished data).

In order to maximise the representativeness of the sample of elderly people who had fallen (rural and urban areas), Toulouse University Hospital and a representative sample of eleven public hospitals within a distance of 155 kms around Toulouse (mostly rural areas) will participate in this study. The RISING-DOM team of each hospital centre consists of a principal investigator geriatrician, a nurse, a Clinical Research Associate (CRA) and associated investigators. Following an EMS home intervention, the EMS staff should provide the potentially eligible population with an information sheet about the RISING-DOM study. Subjects enrolled in this study are identified using the database of the ED Observatory of the Occitanie region (ORU-Occitanie).

### Inclusion and exclusion criteria and recruitment procedure

During the recruitment phase, the ORU-Occitanie makes available to the CRA and investigators participating in this study (via its secured digital professional platform) a daily read-only listing of subjects over 70 years old who have called the EMS because of a fall issue. Calls are automatically screened according to the following key words: fall, fell, fracture, lift, lifted, lifting, on the ground and slipped.

The CRA of each hospital centre is in charge of selecting subjects in his/her territory and ensures that the selected subjects meet the inclusion and exclusion criteria listed in Table 1. Subjects with cognitive and hearing impairments or other disorders are not excluded if they have close relatives or a legal representative able to give consistent phone information. Subjects can be included in the study regardless of the interventions already underway even if they are related to falls prevention. However, subjects with activities of daily living (ADL) set to 0 are excluded, given that PIP intervention would not be appropriate.

**Table 1:** RISING-DOM inclusion and non-inclusion criteria

Inclusion criteria	Exclusion criteria
Age ≥ 70 years	Total dependency (ADL at 0)
Living at home	Entry into a nursing home already scheduled in the next 3 months
Living at maximum 45 min from a hospital centre participating in the study (for logistical reasons)	Subject already enrolled in this study
Intervention of the EMS for a fall at home without there being hospitalisation or with hospitalisation less than 24 h	
Participant (close relatives or legal representative) able to give phone information	
Participant (close relatives or legal representative) has given its consent for participation in the study	
Participant affiliated to a social security scheme	

*EMS* emergency medical service, *ADL* activities of daily living [34]

Each CRA has a nominative account to connect to the ORU-Occitanie platform and can see patient forms with their identity (first name, last name,sex, age), contact details [phone number and identity of the caller (patient, family, other)], place of intervention, date and time of the incident, destination to which the patient was possibly referred to and name and phone number of participant’s GP whenever available and the reason for their calls. In order to check whether the daily ORU-Occitanie forms match the inclusion and non-inclusion criteria, the CRA is in charge of calling each subject and ensuring his/her eligibility to be enrolled in this study. Thereafter, the geriatrician investigator calls back the subject to give all the needed information about the study, obtains his/her oral informed consent and proceeds to his/her randomisation via the RISING-DOM electronic Case Report Form secured website (e-CRF). The actions/interventions planned for each group are detailed in Table 2. RISING-DOM is an open study. The enrolled participants, the investigators and the nurses are informed about the study group allocation after randomisation. However, the CRAs who are in charge of collecting data related to primary and secondary outcomes are not aware of this information in order to maximise data collection objectivity.

**Table 2:** Details on planned actions/interventions in the RISING-DOM compared groups

***Intervention group***	***Control—usual care group***
CGA and fall record carried out at home by an experienced geriatric nurse at the first home visit	
PIP proposal discussed beforehand by a MDTM and with the GP to modify potentially reversible factors	
Commented delivery of PIP for the fall prevention and healthy aging and log-book providing throughout the nurse home visit	Standard recommendations of fall prevention and healthy aging and log-book sent by e-mail or a postal consignment
Nurse follow-up for implementing the PIP and ensuring its effective application or reporting the possible blocking points over the 24-month follow-up period (6 visits and 5 phone calls)	
CRA phone calls over the 24-month follow-up period to collect primary and secondary outcomes	CRA phone calls over the 24-month follow-up period to collect primary and secondary outcomes

*CGA* Comprehensive Geriatric Assessment [35], *PIP* Personalised Intervention care Plan, *MDTM* Multidisciplinary Team Meeting, *GP* General Practitioner, *CRA* Clinical Research Associate

### Participants

Subjects enrolled in this study are over 70 years old, have fallen at home and called an EMS of the territories participating in the study during the inclusion period, without being hospitalised or hospitalised for less than 24 h. The subjects living in nursing homes are excluded from this study (Table 1).

The patient’s oral informed consent is required. However, if the patient is not able to correctly understand the information provided and/or to express his/her wishes, the oral consent should be given to the investigator by close relatives or the legal representative.

The inclusion period started in October 2019 and lasts 30 months. By the end of this period, a total of 1,190 subjects should be enrolled.

### Data collection procedure and randomisation

During the phone call, prior to the enrolment of each participant, the investigator collects information related to the patient’s medical history, his/her current list of pharmacological and non-pharmacological treatment, his/her lifestyle and previous history of falls.

After study recruitment and collection of baseline data, each participant is randomly allocated (1:1) to either the intervention or control arm. Blocked randomisation with random block sizes will be performed. The randomisation will be stratified according to lifestyle (living alone versus living with one’s relatives) and the existence or not of a history of a fall in the previous 12 months and the site.

Data on outcomes (death, hospitalisations, institutionalisation and number of additional EMS calls relating to falls) are collected prospectively by the CRA at 6, 12, 18 and 24 months (M6, M12, M18 and M24 respectively) of the patient’s follow-up and then every six months until the end of the study for both groups. The Activities of Daily Living (ADL) [34] and quality of life (EQ-5D-5L) [36, 37] scales are collected by the CRA once a year at inclusion, 12 and 24 months of follow-up. The exhaustive list of the variables collected by the CRA during the RISING-DOM study for all patients (intervention and control group) is summarised in Table 3. For both groups, a log-book is given to the participants to prospectively collect the events occurring during the follow-up period such as falls, hospitalisations and other side effects with their date. This aims to help the CRA collect with precision the occurrence of events. This document is not accessed/collected by the study team and it works simply as a memory aid. Data collected during nurse home visits (six visits) and nurse phone calls (five phone calls) for the “intervention group” are listed in Table 4.

**Table 3:** RISING-DOM data and timeline collection and for ‘intervention group’ and ‘usual-care control group

	CRA call	Investing call	CRA calls					
		Enrolment allocation	Baseline assessment	Follow-up assessment				
	-t1	t0	M0	M6	M12	M18	M24	^b^
Screening for eligibility	+	+						
***Sociodemographic & medical data***								
Date of birth	+							
Sex	+							
Participant personal data	+							
GP information data	+							
Date, hour and location of fall	+							
Fall circumstances and consequences	+							
Legal protection measures	+							
Education level	+		+					
Life style (living alone or with a partner, family, etc.)		+						
Domestic aid			+					
Medical history		+						
Fall history in the last 12 months		+						
Medical and non-medical treatments		+						
Number of hospitalisations in the last 6 months			+					
***Outcome data***								
Death				+	+	+	+	+
Institutionalisation				+	+	+	+	+
Hospitalisations				+	+	+	+	+
Additional EMS calls relating to a fall^a^				+	+	+	+	+
ADL [34]			+		+		+	
EQ-5D-5L [36, 37]			+		+		+	

*GP* General Practitioner, *CRA* clinical research associate, Investing investigator, *ADL* activities of daily living, EQ-5D-5L EuroQol-5 Dimension-5 levels, M0, M6, M12, M18, M24 match months 0, 6, 12, 18 and 24 respectively from the enrolment (t0) till the end of the follow-up period. ^a^additional EMS calls relating to a fall are collected via the Regional Emergency Department Observatory of Occitanie. ^b^Data on death, hospitalisation and institutionalisation and additional EMS calls are collected by the CRA (M6, M12, M18 and M24) and every six months until the end of the follow-up period of the last enrolled subject

**Table 4:** Participant data collection timeline throughout calls and visit assessments (continued on next page)

	Nurse Visit 0	Nurse Visit 1	Nurse call 2	Nurse call 3	Nurse Visit 2	Nurse call 4	Nurse Visit 3	Nurse call 5	Nurse Visit 4	Nurse call 6	Nurse Visit 5
	≤ 14 d	≤ 30 d	M2	M4	M6	M9	M12	M15	M18	M21	M24
***Socio-demographic and medical data checking***											
Participant contact information checking (address and phone number)	+		+	+	+	+	+	+	+	+	+
Participant's family, legal representative or trusted person contact information checking	+		+	+	+	+	+	+	+	+	+
Legal protection measures checking	+		+	+	+	+	+	+	+	+	+
General Practitioner information data checking	+		+	+	+	+	+	+	+	+	+
Life style checking (living alone or with a partner, family, etc.)	+		+	+	+	+	+	+	+	+	+
Domestic aid checking	+		+	+	+	+	+	+	+	+	+
Medical history checking	+		+	+	+	+	+	+	+	+	+
Pharmacological and non-pharmacological treatments	+		+	+	+	+	+	+	+	+	+
Hospitalisation history from the last contact	+		+	+	+	+	+	+	+	+	+
***Standardised geriatric assessment***											
Body weight and body mass index	+				+		+		+		+
MMSE [38]	+				+		+		+		+
Mini-GDS [39]	+				+		+		+		+
ADL [34]	+				+		+		+		+
IADL [40]	+				+		+		+		+
Fried criteria [33]	+						+				+
SPPB [41]	+						+				+
MNA [42]	+				+		+		+		+
Alcohol consumption	+				+		+		+		+
***Fall record***											
Fall from the last contact	+		+	+	+	+	+	+	+	+	+
Blood pressure	+				+		+		+		+
Check for orthostatic hypotension	+				+		+		+		+
One-leg balance [43]	+				+		+		+		+
Foot examination	+				+		+		+		+
Sensorial assessment	+				+		+		+		+
Amsler Grid [44]	+				+		+		+		+
Pain survey: localisation and intensity	+				+		+		+		+
Sleep survey: sleeping disorders, tiredness upon waking, drowsy episodes during the day, night awakenings	+				+		+		+		+
Environment evaluation: type of habitat, housing occupation status environmental hazards, planned improvements	+				+		+		+		+
Technical walking aid use checking	+				+		+		+		+
Risk taking	+				+		+		+		+
***Summary data and monitoring***											
Monitoring of therapeutic changes					+		+		+		+
Compliance / difficulty of medication intake	+		+	+	+	+	+	+	+	+	+
Emerging of new events (pathologies, social, etc.)		+	+	+	+	+	+	+	+	+	+
PIP presentation and application mode		+	+	+	+	+	+	+	+	+	+
Adherence to recommendations		+	+	+	+	+	+	+	+	+	+
Primary care physician frequentation			+	+	+	+	+	+	+	+	+
Other specialist consultation			+	+	+	+	+	+	+	+	+

*GP* General Practitioner, *d* day, *M* month, *MMSE* Mini Mental State Examination, *Mini-GDS* Mini Geriatric Depression Scale, *SPPB* Short Physical Performance Battery, *ADL* Activities of daily living, *IADL* Instrumental Activities of Daily Living, *MNA* Mini Nutritional Assessment, *PIP* Personalised Intervention care Plan. Nurse visit 0 and 1 should be done within a maximum of 14 days and 30 days after enrolment. M2, M4, M6, M9, M12, M15, M18, M21 and M24 match months 2, 4, 6, 9, 12, 15, 18 and 24 respectively from the enrolment till the end of the follow-up period

### Experimental design

An intervention includes three main actions: (*i*) CGA [35] and fall risk factor assessment, (*ii*) PIP to address potentially reversible and modifiable factors and (*iii*) patient follow-up by an experienced geriatric nurse over the 24-month follow-up period (Table 2).

(*i*) The CGA is performed during the first nurse visit at the participant’s home (V0) within a maximum of 14 days from the date of the EMS intervention. In addition, a fall record is performed to track potential risk factors (*e.g*. sensorial assessment, one-leg balance [43], Amsler grid [44], environmental hazards, etc.). Details on performed assessments and data collected throughout the nurse home visit are provided in Table 4.

(*ii*) A PIP is established throughout a Multidisciplinary Team Meeting (MDTM) within a maximum of 10 days following the first nurse visit. The geriatrician investigator, the nurse and the hospital pharmacist participate in this meeting to discuss the clinical and social status of the participant and suggest appropriate solutions regarding possible warning signs.

The PIP is proposed according to emphasis on preventing falls, standard geriatric recommendations [45], medical treatment adjustment and takes into account the patient's desires and concerns. It is discussed and approved beforehand by the participant’s GP and then given to the participant during the nurse visit at home within 10 days after the MDTM (V1). A maximum of three recommendations are proposed to the participants to maximise their adherence. For instance, prescription of physically adapted activities, attending of equilibrium and fall prevention workshops offered by the municipality, nutrition education, medication review carried out in a primary care setting, etc. The inclusion of the GP in the proposed intervention program should ensure patient adherence and compliance.

During the next four visits (V2, V3, V4, V5) every 6 months, the nurse re-evaluates the patient and ensures the effective application of the PIP or reports the possible blocking points. Relevant points are addressed after each visit to the participant’s GP. In addition, five nurse phone calls are scheduled at 2, 4, 9, 15 and 21 months (Fig. 1) in order to keep in touch with the participant and follow the PIP application.

In case of a health crisis and/or if it is impossible for the nurse to visit the participant’s home, the nurse can exceptionally perform a remote screening via video call or telephone if the subject does not have the necessary IT equipment or is unable to use it.

## Outcomes

Primary and secondary outcome data are collected by CRA phone calls twice a year throughout the study. The CRA is blind of the participant group. The end of the patients’ follow-up matches the end of the follow-up of the last enrolled participant. This means that the first enrolled participant has 54 months of follow-up (compared to 24 months for the last enrolled participant). In case of difficulty of reaching the elderly, the CRA contacts: 1- the patient’s close relatives or legal representative and 2- the participant’s GP in order to collect outcome measures.

### Primary outcome

The primary outcome in this study is time to institutionalisation or death (before institutionalisation) in both groups (first occurring event). It is a composite criterion that consists of the period (days) between the enrolment (t0) until the onset of institutionalisation or death before institutionalisation and that is collected every 6 months. It is considered as an appropriate indicator of the intervention efficiency for the target population based on our pilot feasibility study.

### Secondary outcomes

Secondary outcomes are collected at inclusion, 12 and 24 months follow-up and are as follows:

- **Functional dependency level**
  The ADL scale is commonly used to assess basic functional capacities [34]. The scale stands on a score of 6 related to the execution of basic tasks of daily living (bathing, dressing, using the toilet, getting into or out of a bed or chair, faecal and urinary continence and eating).
- **Quality of life assessment**
  The EuroQol-5 Dimension-5 levels (EQ-5D-5L) is a generic tool to measure the health-related quality of life on five dimensions (mobility, self-care, usual activities, pain/discomfort and anxiety/depression) [36, 37]. Patients are also asked to give a score out of 100 about their global quality of life (health analogue scale).
- **Rate of additional EMS calls relating to a fall over 24 months**
  These data are collected from the ORU-Occitanie platform and verified during the CRA follow-up phone calls.
- **Time before occurrence of the first additional EMS call relating to a fall**
  This represents the time between the first fall recorded at enrolment time (t0) and the occurrence of the first additional EMS call relating to a fall over 24 months.
- **Unscheduled hospitalisation rate over 24 months**
- **Time before occurrence of the first unscheduled hospitalisation**
- **Death and institutionalisation rate over 24 months**

### Sample size

The 24-month event rate (institutionalisation or death before entering an institution) is estimated at 18% in the control arm based on available data in the literature [46]. A relative reduction of 30% is expected in the intervention arm at 24 months (Hazard Ratio = 0.679) with a two-sided test. 211 events should detect this Hazard Ratio (with a two-sided test Log-rank test) with a power of 80% and alpha risk of 0.05. With a study over 54 months, a uniform inclusion in the first 30 months and 10% of people lost to follow-up per year, it will be necessary to include 595 subjects per group to reach this number of events, thus 1,190 subjects in total.

### Statistical methods

A detailed analysis plan will be defined and will be validated by the Scientific Council of the study. Subsequent modifications must be made before the database freeze and will be systematically approved by the Scientific Council.

The consistency of the data will be checked using logic checks and the database will undergo the necessary revisions before being declared frozen.

The statistical analysis will be conducted using SAS software version 9.4 (SAS Institute Inc., Cary, NC) or a later version. The significance threshold is set at 0.05 and all the tests will be performed with a two-sided test.

A descriptive analysis of the entire recruited population will be carried out to verify whether there are any deviations from the protocol at the time of inclusion. The quantitative variables will be expressed as mean ± standard deviation, medians and interquartile ranges. The qualitative variables will be expressed in numbers and percentages. At inclusion, the initial characteristics of the participants (socio-demographic characteristics, history of falls, ADL, EQ-5D-5L) will be described in both arms. The balance of randomisation will be checked by strata. The existence of a hospital centre effect will be explored.

### Primary outcome analysis

For the primary outcome, an intention to treat analysis will be conducted and will compare time to institutionalisation or death (before institutionalisation) between the two groups using a two-sided log-rank test in accordance with the sample size of the study. The subjects will be censored in case of an event occurrence or loss to follow-up. A sensitivity analysis will be conducted by a multi-variable survival model (with a Cox proportional-hazards model in the event that the risk proportionality hypothesis is verified) in order to take into account the stratification parameters and potential identified confounding factors (i.e. age and ADL). Marginal models with a robust variance–covariance matrix or sandwich estimator will be considered. Additional sensitivity analyses will be conducted taking into account the subjects who have been institutionalised (*i.e.* people placed in a nursing home) or who have already died at the time of their missed follow-up. The raw and adjusted hazard ratios will be expressed with their confidence interval set at 95%.

### Secondary outcomes analysis

The analysis of the secondary outcomes are conducted according to *(i)* a similar approach to that described for the analysis of the primary outcome for the criteria: time to the first additional EMS call relating to a fall and time to the first unscheduled hospitalisation *(ii)* the mean number of EMS calls relating to a fall, the mean number of unscheduled hospitalisations and the percentage of deceased or institutionalised subjects at 24 months will be compared between the two groups by generalised linear multi-variable models adjusted on the stratification and the potential confounding factors (age and AD, in particular) *(iii)* the comparison between the two groups of the level of the functional autonomy (ADL scale) and the quality of life ( EQ-5D-5L scale) at 24 months will be carried out by generalised multi-variable linear models adjusted to stratification and to the potential identified confounding factors (age) and the level of the score (ADL or EQ-5D-5L at inclusion) if appropriate.

Additional per protocol analysis will be conducted in the population of subjects who have complied with the protocol (in the intervention arm having accepted at least 80% of all visits and calls proposed during follow-up and for whom a PIP will have been proposed).

## Discussion

As the population is aging, the number of frail elderly people and those who are living with multiple chronic conditions is increasing [47, 48]. Most of them usually prefer to age in place [49], but falls remain a strong predictor of both placement in a skilled-nursing facility and death [5, 50].

About a third of community‐dwelling people over 65 years old fall each year [51]. Falls among elderly people have a major impact on quality of life and are a financial burden to health care systems [50, 51] A number of epidemiological studies have identified the risk factors of falling and highlighted the responsibility of multiple interacting factors for the majority of falls [52]. Some preventive intervention programs have been tested and assessed in different target people among various countries [53–57]. Guidelines for preventing falls advocate approaches based on comprehensive risk assessment (including environmental evaluation) and exercise programs. However, most of these intervention programs are not addressed in primary health care [29] and are difficult to implement in ED [14, 58, 59].

The RISING-DOM project has emerged from an insistent request of our EMS department that has warned about the growing number of calls from elderly people who have fallen with no intervention or specific follow-up. In addition, many of them have had multiple fall-related calls, sometimes within a short period of time, which is in accordance with previous studies [60, 61]

These calls may, however, be early indicators of problems requiring comprehensive medical evaluation and follow-up [62]. The main purpose of the RISING-DOM study is to assess the effectiveness of a PIP monitored by a close nurse home follow-up on time to institutionalisation or death. The quality of life, the functional autonomy evolution, the number of unscheduled hospitalisations, number of EMS calls relating to a fall, the number of deaths and institutionalisations are also monitored as secondary outcomes. The originality of this study is to build a professional network around a poorly studied and vulnerable population to prevent institutionalisation and death, and help to define the best approach for fall management based on home health care continuity. Our research network covers rural, as well as urban and semi-urban communities. If this protocol and the new organisation proposition reveal their efficiency, it could be easily generalised to other regions. During the first COVID-19-related national lockdown, (from 17 March 2020 to 11 May 2020), planned nurse home visits of follow-up (*n* = 7) have been performed remotely and have been appreciated by the elderly, as they felt isolated. However, the recruitment of new participants was interrupted in that period and has gradually resumed according to the implication of the study sites in the management of the health crisis. Consequently, we might encounter some difficulties to reach the expected sample size. In that case, a substantial modification will be submitted in a new protocol study version. The first results should be available in 2024.

## Data Availability

The data that support the findings of this study are available from the Toulouse University Hospital but restrictions apply to the availability of these data, which were used under license for the current study, and so are not publicly available. Data are however available from the authors upon reasonable request and with permission of the Toulouse University Hospital.

## Abbreviations

ADL: Activities of Daily Living
CGA: Comprehensive Geriatric Assessment
CRA: Clinical Research Associates
e-CRF: Electronic Case Report Form
GP: General Practitioner
ED: Emergency Department
EMS: Emergency Medical Service
EQ-5D-5L: EuroQol-5 Dimension-5 levels
IADL: Instrumental Activities of Daily Living
Mini-GDS: Mini Geriatric Depression Scale
MMSE: Mini Mental State Examination
MNA: Mini Nutritional Assessment
MDTM: Multidisciplinary Team Meeting
ORU-Occitanie: Observatory of Occitanie region
PIP: Personalised Intervention care Plan
SPPB: Short Physical Performance Battery
